# Previous dry socket as a risk factor for alveolar osteitis: A nested case-control study in primary healthcare services

**DOI:** 10.4317/jced.59586

**Published:** 2022-06-01

**Authors:** Maria Taberner-Vallverdú, Octavi Camps-Font, Cosme Gay-Escoda, Maria-Angeles Sánchez-Garcés

**Affiliations:** 1 DDS. University of Barcelona, Barcelona (Spain); 2 Associate Professor of Oral Surgery. Professor of the Master Degree Program in Oral Surgery and Implantology, School of Dentistry, University of Barcelona, Barcelona (Spain); 3 MD, DDS, PhD, MS, EBOS, OMFS. Chairman and Professor of Oral and Maxillofacial Surgery, School of Dentistry, Barcelona. Director of the Master Degree Program in Oral Surgery and Implantology (EHFRE International University/ FUCSO). Coordinator/Researcher of the IDIBELL Institute. Head of the Department of Oral Surgery, Implantology and Maxillofacial Surgery, Teknon Medical Center, Barcelona (Spain); 4 MD, DDS, PhD, MS, EBOS. Lecturer in Oral Surgery. Professor of the Master Degree Program in Oral Surgery and Implantology, School of Dentistry, University of Barcelona. Researcher of the IDIBELL Institute, Barcelona (Spain)

## Abstract

**Background:**

Dry socket is one of the most common complications following tooth extraction, though no studies have been made on its main risk factors in the primary healthcare services of Barcelona (Spain). Objectives: To analyze the influence of different factors upon the appearance of dry socket in patients attended in the primary care setting, and to determine the possible presence of risk factors in patients who have suffered a previous episode of dry socket.

**Material and Methods:**

During 24 months, questionnaires were filled with data on the patients seen in different public primary healthcare services in the area of Barcelona (Spain). A case-control study was conducted to identify the main risk factors for developing complications in the form of dry socket.

**Results:**

A mandibular location of the extracted tooth, poor oral hygiene, difficult extraction, and previous dry socket increased the risk of developing this complication. In patients with dry socket in the past, the risk of developing the same complication again, adjusted for difficulty of extraction, was seen to increase 11.45-fold (OR: 11.45; 95%CI: 1.06 to 123.74; *p* = 0.045).

**Conclusions:**

The risk factors for dry socket are a mandibular location of the extracted tooth, poor oral hygiene, difficult extraction, and particularly a history of dry socket in the past. The identification of these factors the prevention of dry socket in each patient could be improved.

** Key words:**Dry socket, risk factors, extraction, complications.

## Introduction

Conventional or surgical dental extractions are a very common treatment in dentistry. Healing of the extraction socket depends on local and systemic factors, and complications in the healing process can have an impact upon patient quality of life (1). One of the possible complications is dry socket, which may be so painful that it can threaten different aspects of daily life such as eating and work, etc. Moreover, dry socket is a challenge for clinicians, since there is no consensus on the best treatment strategy for this disorder.

The incidence of dry socket in epidemiological studies is variable, and no research into its main risk factors has been carried out in our setting. The present study was designed to identify the influence of different factors upon the appearance of dry socket in patients attended in the public healthcare system, where dental extractions are performed daily.

Dry socket may develop when the post-extraction alveolus is prematurely disintegrated, leaving the bone unprotected and exposed to the oral environment. Both bacteria and food can fill the socket, and their degradation products are believed to lead to greater dissolution of the clot (2). Thus, the essential features of dry socket are loss of the blood clot in the socket, together with exposure of the bony walls, sensitivity on probing and occasionally also fever (3).

Other less common terms used to refer to this condition alveolar osteitis, postoperative alveolitis, alveolalgia, fibrinolytic alveolitis (4), painful dry socket or localized dry socket (5).

The prevalence of dry socket as reported in the literature varies between 3-4% following conventional extractions, though the condition is more frequently diagnosed after the surgical removal of impacted third molars, where the risk increases up to 10-fold (6), with a prevalence of 25-35% of all surgical extractions (7).

Although the pain associated with dry socket is self-limiting, the disorder causes considerable problems for both patients (8) and healthcare professionals, in view of the intensity of the pain (9) and the need for more postoperative visits (10).

Apart from the relation to fibrinolysis, the etiological mechanism underlying dry socket remains unclear (11,12). Nevertheless, some systemic risk factors have been identified, such as the medication used by the patient, along with other local factors such as oral hygiene or the type of anesthesia used during tooth extraction (13).

Other factors contributing to the occurrence of dry socket are traumatic extractions, the female gender, tobacco use, oral contraceptive use and pre-existing infections (14). The latest published studies on this subject, involving the use of microbiological techniques, suggest that the presence of certain pathogens may be implicated in the origin of dry socket, since patients who develop alveolar osteitis after dental extraction present a microbiota different from that seen in patients without postoperative complications (15,16). In addition, it must be noted that a previous history of dry socket has been associated to the risk of developing the complication again in future dental extractions (17,18). The use of next-generation sequencing techniques can be a big help in confirming the role of the microbiota in the development of alveolar osteitis.

The evaluation of risk factors associated to dry socket is a noninvasive preventive strategy with no cost for either the patient or the professional that can allow the identification of individuals at risk of developing this complication. Accordingly, the present study was carried out to identify the main risk factors for dry socket in the primary healthcare services of Barcelona (Spain).

## Material and Methods

-Study design

A nested case-control study was carried out with an initial cohort of 9156 patients consecutively attended during the period between October 2017 and September 2019 in 8 centers of the Catalan Public Healthcare System in the metropolitan area of Barcelona. The design of the study followed the Strengthening the Reporting of Observational Studies in Epidemiology (STROBE) declaration for case-control studies (19). The protocol met the guidelines of the Declaration of Helsinki, and was approved by the Institutional Review Board of Atenció Primària Jordi Gol (IDIAP Jordi Gol), as well as by the directors of the different collaborator centers (Ref. P17/140).

Patients were given full information about the surgical procedures and treatment alternatives, and written informed consent was obtained in all cases. The preoperative analysis included clinical and radiographic examinations (periapical or panoramic radiographs).

-Eligibility criteria

All patients who underwent tooth extraction, without restrictions referred to the location of the tooth, were included in the study. Subjects under 18 years of age were excluded.

The dry socket or alveolar osteitis group (AO) comprised tooth extraction in patients that presented moderate to severe postoperative pain with an onset at least 48 hours after the surgical procedure. These cases had an empty socket and presented no apparent signs of infection (no suppuration). The control group (CG) in turn was composed of patients who underwent the same surgical procedure within the abovementioned time frame but did not develop dry socket during the postoperative period (proportion 1:2). The diagnosis of AO was made by the same clinician who performed the procedure, based on clinical criteria. In order to avoid wrong classification bias, patients with incomplete clinical records were excluded from the analysis.

-Definition of outcome variables

The AO group included those patients with loss of the blood clot in the socket together with exposure of the bone walls and sensitivity upon probing (3). The diagnosis of AO was made based on the following parameters: the presence of denuded socket, a foul smell, and intense pain at the extraction site.

-Surgical procedure

Dental extractions were performed by clinicians from the Institut Català de la Salut (Catalan Public Healthcare System), with extensive professional experience (18-25 years). Each clinician performed the procedure according to his or her personal expertise. Owing to the retrospective nature of the study, minor variations in surgical technique could have occurred.

Postoperative instructions were provided, and the use of the prescribed drugs was explained by means of a paper sheet given to the patient. Patient compliance was not specifically assessed.

-Data sampling

A single researcher (MT) compiled the patient data collection sheets and examined all the clinical records. The data retrieved were age, gender, current medication, smoking habits (number of cigarettes/day), history of infection or pericoronitis, oral hygiene (good, regular or poor), surgical variables (tooth extracted, date of extraction, local anesthetic, bone removal, tooth sectioning and surgeon experience), antibiotic and antiseptic administration, and intraoperative complications. The postoperative follow-up was also registered (symptoms onset, type of treatment, number of visits, number of analgesic Tablets).

The clinician who performed tooth extraction was the same who filled the data collection sheets, which were periodically delivered to the researcher.

-Statistical analysis

Calculation of the sample size was based on the assumptions that dry socket affects up to 5% of all patients (20) and that a previous history of dry socket increases the risk of this complication at least 10-fold (17,18). Considering an α risk of 0.05, a statistical power of 80% and a 10% dropout rate, a total of 17 patients with dry socket were required. Two controls were included for each patient with dry socket (i.e., 34 controls were analyzed) in order to increase the statistical power without recruiting more cases.

The patient characteristics were reported as absolute and relative frequencies for categorical outcomes. A bivariate analysis using the chi-square test or Fisher’s exact test (as required) was performed to compare the groups. The odds ratio (OR) with 95% confidence interval (95%CI) were calculated for each covariable.

**Figure 1:**
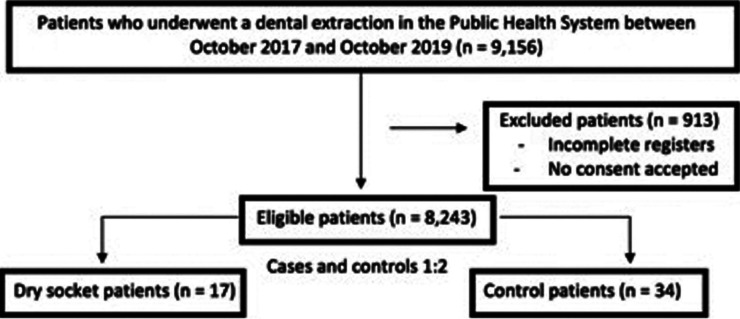
Flow diagram of the study participants.

Normal data distribution (patient age) was explored using the Shapiro-Wilk test and through visual analysis of the P-P plot and box plot. Where normality was rejected, the interquartile range (IQR) and median were calculated. Where distribution was compatible with normality, the mean and standard deviation (SD) were used. Parametric and nonparametric tests (independent t-test or Mann-Whitney U-test) were used to explore the association of age to alveolitis (Fig. 1).

Multivariate analysis was performed using non-conditional binary logistic regression to assess the influence of a previous history of dry socket upon the likelihood of suffering this condition again (Table 1). Consequently, after adjusting for potential confounders and effect modifiers, the adjusted effect of the exposure variable (i.e., a previous history of dry socket) was measured. The initial model included all the variables with a *p-value* < 0.30 in the bivariate analysis. Then, all possible submodels that could be formed in compliance with the hierarchical norm were estimated. The reduced model was rejected if the change of the β adjusted coefficient exceeded 10%. Thus, the simplest model was selected if this difference was under 10%, limiting the number of independent covariables to a maximum of two in order to avoid over-fitting of the model.

**Table 1:**
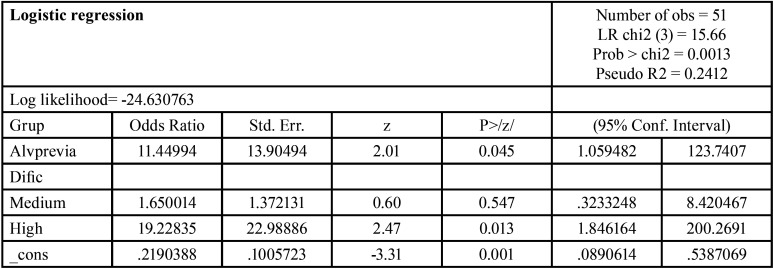
Results of the logistic regression model.

The statistical analyses were carried out using the Stata14 package (StataCorp®, College Station, USA). Statistical significance was considered for *p* < 0.05 in all comparisons.

## Results

Seventeen dry sockets (in 17 patients) were recorded (Fig. 1). The patient-based prevalence was 0.19% (95%CI: 0.12 to 0.30).

The bivariate analysis revealed a significant association between dry socket and mandibular location (OR = 4.40; 95%CI: 1.09 to 19.44; *p* = 0.017), poor oral hygiene (OR = 17.14; 95%CI: 1.16 to 252.91; *p* = 0.015), difficult tooth extraction (OR = 22.29; 95%CI: 1.49 to 333.05; *p* = 0.004) and a previous history of dry socket (OR = 13.75; 95%CI: 1.27 to 669.26; *p* = 0.012).

The mean patient age was 51.75 years (SD = 15.03) and 51.95 years (SD = 18.48) in the AO and CG groups, respectively (MD = -0.20; 95%CI: -10.60 to 10.21; *p* = 0.97).

After adjusting for difficulty of dental extraction, the effect of a previous history of dry socket on the likelihood of suffering this condition again was 11.45 (ORa = 11.45; 95%CI: 1.06 to 123.74; *p* = 0.025) (Table 2). The change in the likelihood ratio of the logistic regression model was statistically significant (ꭓ2 = 15.66; degrees of freedom (df) = 3; *p* = 0.001). The Nagelkerke R2 value was 36.7% and thus explained nearly one third of the observed variation. Despite the small number of cases, the Hosmer-Lemeshow test showed good data fit (*p* = 0.96), and there was no colinearity (tolerances ≈ 1.00; variance inflation factors ≈ 1.00). The assumptions of the model were fulfilled.

**Table 2:**
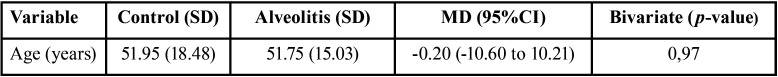
Results of the bivariate analysis of the scale variables.

## Discussion

The prevalence of dry socket found in our study was lower than the values published in the literature, probably due to the difficulty of patient traceability in the public healthcare system, since some subjects may not have returned to the office despite the painful disorder.

According to the scientific literature, those patients with a previous history of dry socket show a greater predisposition to develop a new episode of this complication (17,18). This agrees with our own observations, and has been described in epidemiological studies of dry socket as a statistical finding, though the cause of this greater predisposition remains unclear (Table 3). Recent articles suggest that the presence of bacteria in the post-extraction socket may play a key role as a risk factor for dry socket (15); this in turn could justify the results obtained in our study.

**Table 3:**
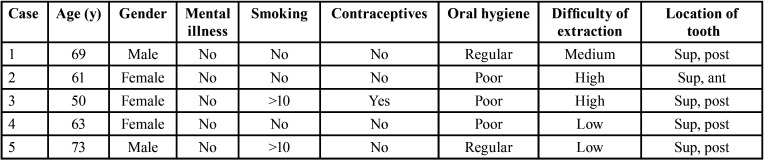
Main demographic characteristics of the patients with a history of dry socket.

As a modifying factor, Chuang *et al*. (21) pointed to the evidence of preexisting infection, which contributes to inoculate microorganisms in the exposed socket after tooth extraction, and therefore increases the risk of developing dry socket (22). Regarding the main microorganisms associated to dry socket, Partharsarathi *et al*. (23) reported that extractions performed for periodontal reasons have a 7.5-fold increase in relative risk of developing dry socket - a fact that could be conditioned by the kind of pathogens involved. Nevertheless, one study documented a statistically significant increase in the incidence of dry socket in tooth extractions performed for orthodontic indications versus the presence of pericoronitis (24). The data obtained in our study are inconclusive in this regard, because no bacterial samples were obtained, though poor oral hygiene was indeed identified as a relevant risk factor in our series (*p* = 0.015, OR 17.14) (Table 4,  Table 4 cont.), and this could lead to conclusions about the kind of bacteria involved.

**Table 4:**
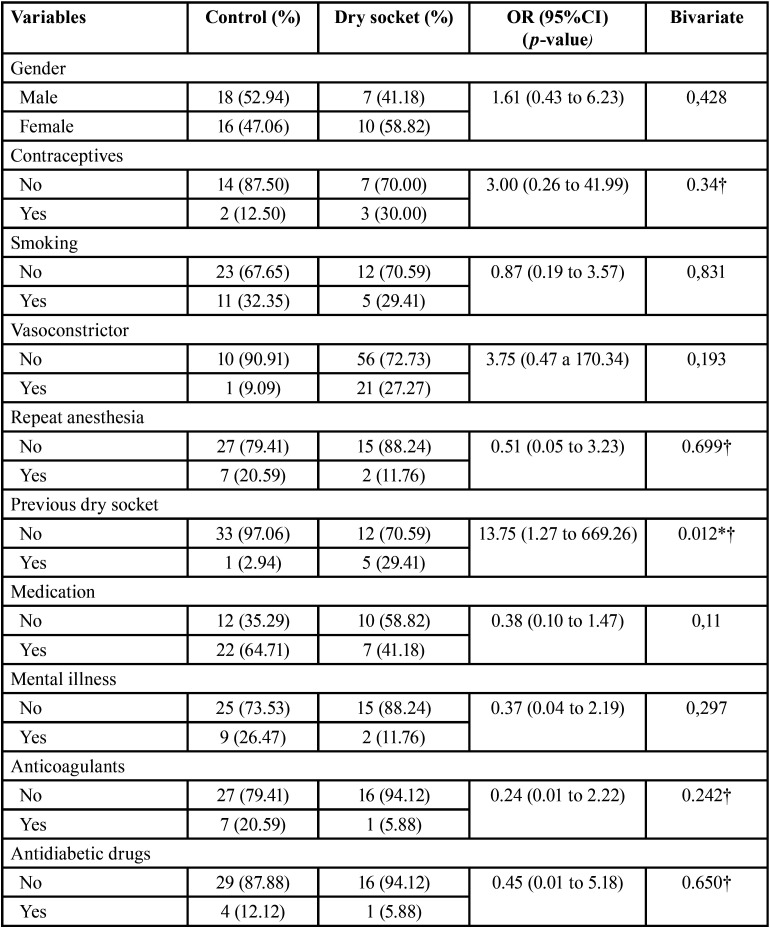
Results of the bivariate analysis of the categorical variables.

**Table 4 cont.**
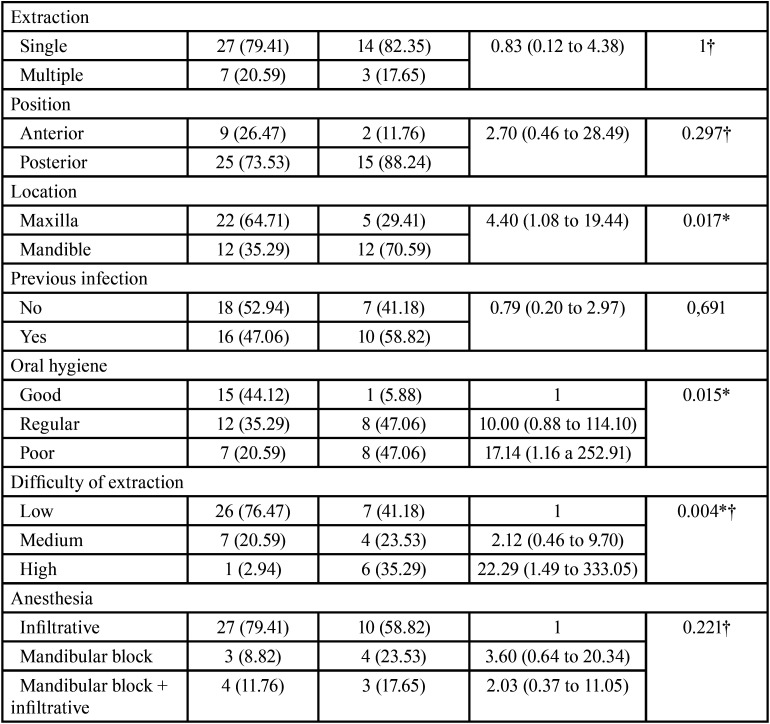
Results of the bivariate analysis of the categorical variables.

In a recent study comparing the microbiota present in post-extraction alveoli with and without dry socket, only 45 of the 151 bacterial species identified were found to be common in both groups, which may suggest a bacterial role in the development of dry socket (15). The most frequently found bacteria in the sockets were: *P. nanceiensis, A. odontolyticus, T. maltophilum, V. dispar, T. forsythia, L. mesenteroides*, *P. intermedia, P. melaninogenica, P. micra and F. nucleatum* – most of these species being gramnegative. In this study the time lapse between tooth extraction and sample collection was similar in both groups. However, while the controls presented no postoperative complications, the patients in the AO group had pain as assessed by a visual analogue scale (VAS), with an onset at least 48 hours after the surgical procedure and an empty socket with no suppuration.

The new sequencing techniques that allow the conduction of metagenomic studies represent an important advance in dry socket research, since many of the risk factors described so far only reflect statistical findings - with the limitations this implies. In recent years, two studies have collected bacterial samples in patients undergoing dental extractions in order to analyze differences in composition of the microbial community in normal healing sockets and dry sockets (15,16).

Our observation of a greater risk of dry socket in extractions in the mandibular zone is consistent with the findings of Oginni *et al*. (13), and is probably due to the greater bone density in the mandible. Also, the difficulty of tooth extraction has again been evidenced as a predisposing factor, since trauma favors delayed healing through compression of the bony lining of the socket, thrombosis of the underlying vessels, reduced tissue resistance, and a predisposition to wound infection (13,22,25-27). In fact, the highest incidence of dry socket has been reported after the surgical extraction of lower third molars, with a 30% greater probability of developing this complication (28).

Regarding the other variables recorded, such as tobacco or contraceptive use, medication, age and gender of the patient, no correlation to the development of dry socket was found. This is consistent with the existing studies, where no consensus has been found regarding such factors as clear modifiers of the risk of alveolar osteitis (23,26).

Our study has some limitations, particularly its limited sample size, which may be explained by the low prevalence of the complication and probably also by the difficulties referred to patient traceability in the public healthcare system. Furthermore, the fact that different clinicians collaborated in recruiting the cases and in collecting the study variables implies that subjective approaches with different criteria may have been involved, despite the instructions given to the clinicians before starting the study. Nevertheless, no previous studies have been conducted in the public care setting on the prevalence of dry socket. It is now possible to confirm the low incidence of this complication and its main risk factors in different public centers with experienced dentists.

Future studies are needed to correlate clinical dry socket to different bacterial species, establishing comparisons with uncomplicated postoperative sockets, and to determine whether there are any differences in bacterial profile among patients that suffer dry socket more than once.

## Conclusions

Within the limitations of this study, it can be concluded that the main risk factors for dry socket are a mandibular location of the extracted tooth, poor oral hygiene, difficult tooth extraction, and particularly a previous history of dry socket. In patients presenting this latter condition, the risk of developing the same complication again, adjusted for difficulty of extraction, increases 11.45-fold.

Since the prevention of dry socket is easier and more cost-effective than its treatment, the identification of such factors may contribute to establish preventive methods in each patient and thus minimize the risk of developing postoperative complications of this kind.
